# Glial and Neuronal Neuroglian, Semaphorin-1a and Plexin A Regulate Morphological and Functional Differentiation of *Drosophila* Insulin-Producing Cells

**DOI:** 10.3389/fendo.2021.600251

**Published:** 2021-07-01

**Authors:** Jason Clements, Kurt Buhler, Mattias Winant, Veerle Vulsteke, Patrick Callaerts

**Affiliations:** Laboratory of Behavioral and Developmental Genetics, Department of Human Genetics, KU Leuven, Leuven, Belgium

**Keywords:** *Drosophila*, insulin-producing cells, glia, morphological and functional differentiation, neuron, cell adhesion molecules, axon guidance molecules, stress resistance

## Abstract

The insulin-producing cells (IPCs), a group of 14 neurons in the *Drosophila* brain, regulate numerous processes, including energy homeostasis, lifespan, stress response, fecundity, and various behaviors, such as foraging and sleep. Despite their importance, little is known about the development and the factors that regulate morphological and functional differentiation of IPCs. In this study, we describe the use of a new transgenic reporter to characterize the role of the *Drosophila* L1-CAM homolog Neuroglian (Nrg), and the transmembrane Semaphorin-1a (Sema-1a) and its receptor Plexin A (PlexA) in the differentiation of the insulin-producing neurons. Loss of Nrg results in defasciculation and abnormal neurite branching, including ectopic neurites in the IPC neurons. Cell-type specific RNAi knockdown experiments reveal that Nrg, Sema-1a and PlexA are required in IPCs and glia to control normal morphological differentiation of IPCs albeit with a stronger contribution of Nrg and Sema-1a in glia and of PlexA in the IPCs. These observations provide new insights into the development of the IPC neurons and identify a novel role for Sema-1a in glia. In addition, we show that Nrg, Sema-1a and PlexA in glia and IPCs not only regulate morphological but also functional differentiation of the IPCs and that the functional deficits are likely independent of the morphological phenotypes. The requirements of *nrg*, *Sema-1a*, and *PlexA* in IPC development and the expression of their vertebrate counterparts in the hypothalamic-pituitary axis, suggest that these functions may be evolutionarily conserved in the establishment of vertebrate endocrine systems.

## Introduction

The insulin-producing cells (IPCs) are a cluster of 14 neurosecretory neurons located in the pars intercerebralis of the *Drosophila* brain. The IPCs are the major source of the *Drosophila* insulin-like peptides (Dilps). They express Dilp2, -3, and -5, which are secreted into the circulatory hemolymph *via* contacts on the aorta (1). Loss of IPCs or IPC function results in insulin/insulin-like growth factor signaling defects, which in turn affect numerous processes, including energy homeostasis, growth and body size, stress resistance, lifespan, fecundity, and behavior (2, 3)

Despite the importance of the IPC neurons, very little is known about their development. While the lineage of the IPCs and the factors and pathways contributing to their specification have been well-described (4, 5), the factors that regulate the morphological and functional differentiation of the IPCs remain almost completely unknown. We have previously demonstrated that the transcription factor Eyeless is required both for the proper differentiation of the IPC neurons as well as the expression of *dilp5* (6). It was later shown that the transcription factor Dachshund also regulates *dilp5* transcription *via* an interaction with Eyeless, but no defects in IPC differentiation were reported in *dachshund* loss-of-function animals (7). A study to identify factors contributing to insulin-like peptide production identified a role for Unc-104 and Rab1 in IPC development, where loss of either protein resulted in larvae with reduced IPC cell number and aberrant IPC morphology (8).

Important classes of molecules in the regulation of neuronal morphogenesis are cell-adhesion molecules (CAMs) and axon guidance molecules (9–11). Glia are closely associated with neurons and exercise significant control over their development, yet our knowledge of the mechanistic basis thereof is far from complete (12, 13). The L1-type cell-adhesion molecules are a family of evolutionarily conserved CAMs important for axonal pathfinding and synaptogenesis during nervous system development in vertebrates and invertebrates (10, 14). Mutations in these genes lead to severe neural defects in various species (15–17). In *Drosophila*, the L1-CAM homolog Neuroglian (Nrg) is expressed in neuronal and glial cells. Elav-dependent differential splicing results in the neuron-specific isoform Nrg-180, while the more broadly expressed Nrg-167 isoform is found in glial cells (18–21). We and others have previously shown that *nrg* and *Semaphorin-1a* (*Sema-1a*) interact genetically (22, 23). Sema-1a, a transmembrane glycoprotein, and its receptor Plexin A (PlexA) act in pathfinding, where both Sema-1a and PlexA can serve as receptor (24–32).

Here we show that Nrg, Sema-1a, and PlexA are required for IPC differentiation. *nrg* loss-of-function results in IPCs exhibiting defasciculation in the median bundle, neurite pathfinding defects resulting in ectopic branches, and aberrant outgrowth of the lateral and subesophageal neurites. We also show that the ectopic branches are the result of loss of Neuroglian function in glia, and that Sema-1a and its receptor PlexA are also required in this process of neuritogenesis. In addition, we find evidence for a possible role of Nrg in the recruitment or organization of synaptic protein complexes in IPCs. Furthermore, we demonstrate that Nrg, Sema-1a, and PlexA not only regulate morphological but also physiological differentiation of IPCs.

## Materials and Methods

### Transgenes and *Drosophila* Stocks

The following fly stocks were used: Canton-S (CanS, wildtype), *nrg^849^* and *nrg^892^* (23, 33), *nrg^GFP^* [also known as *nrg^G00305^*, Bloomington Stock Center (34, 35)], *nrg^3^* (36), *nrg^1^* [Bloomington Stock Center (36)], UAS-*nrg* RNAi [Vienna *Drosophila* RNAi Center, VDRC #6688; validated in (23)], UAS-Nrg-180 and UAS-Nrg-167 (23, 37), 30B07-Gal4 [Bloomington Stock Center (38)], Dilp2-Gal4 (1), repo-Gal4 (39), UAS-DenMark (40), UAS-mCD8-GFP (Bloomington Stock Center), PlexA^GFP^ [PlexA^YD0269^ (41)], Sema-1a^GFP^ [Sema-1a^CA07125^ (42)], UAS-Sema-1a (gift from Corey Goodman), UAS-Sema-1a RNAi [Bloomington Stock Center, TRiP.HMS01307; validated in (43)], UAS-PlexA RNAi [Bloomington Stock Center, TRiP.HM05221; validated in (43)], UAS-mCherry RNAi (Bloomington Stock Center, TRiP VALIUM20-mCherry, attP2) and Dilp2-DenMark (this study).

Dilp2-DenMark (D2:DM), in which DenMark (40) was expressed under the direct control of the *dilp2* enhancer (1) was used to visualize the IPC neurons and their neurites. The p{Dilp2-DenMark} construct was generated by digesting pPTGAL (44) with PstI and removing the portion of the plasmid containing the Gal4 and the polyadenylation sequences. The *dilp2* enhancer, which was generated by PCR amplification of wildtype genomic DNA using the dilp2-enhancer-F and dilp2-enhancer-R primers, was then cloned into the StuI and KpnI sites within the multiple cloning site of the truncated pPTGAL. Next, the PstI fragment containing the multiple cloning site and the polyadenylation sequence from pUAST (45) was cloned into the PstI site approximately 150 bp downstream of the previously cloned *dilp2* enhancer, creating p{Dilp2}. To generate the p{Dilp2-DenMark} construct, DenMark cDNA was obtained by PCR amplification of p{UAS-DenMark} (gift from Bassem Hassan) using the DenMark-F and DenMark-R primers and cloned into the BglII and XbaI sites of p{Dilp2}.

The sequences of the PCR primers used for cloning are as follows:

dilp2-enhancer-F: 5’- CTAAGGCCTCCAACACACACACATTCATTACCCA;
dilp2-enhancer-R: 5’- CTAGGTACCTGGTTATGGGTTTACTGCTTAGGTTG;
DenMark-F: 5’- CTAAGATCTGCCACCATGCCGGGGCCTTCGCCAGGGCT;
DenMark-R: 5’- CTATCTAGATTATCAGGAAGATGTCAGCTGGATAG

### Immunohistochemistry

Whole-mount larval and adult brain immunohistochemistry was performed as previously described (6, 46).

The following primary antibodies were used: mouse anti-GFP (Roche) was used at 1:500, mouse anti-GFP monoclonal antibody 8H11 (Developmental Studies Hybridoma Bank) was used at 1:200, rabbit anti-dsRed (Clontech) was used at 1:500, rabbit anti-Dilp2 (1) was used at 1:1000, rabbit anti-Dilp3 (46) was used at 1:500, rabbit anti-Dilp5 (46) was used at 1:200. Secondary antibodies conjugated to either FITC or Cy3 were used at 1:200 (Jackson ImmunoResearch).

Quantification of the large DenMark-positive densities in the subesophageal region of the adult insulin-producing neurons was obtained by the analysis of confocal stacks with ImageJ (47) and Imaris software (version 6.1.0; Olympus). Images of the immunostained brains were scanned in a single sitting using the same confocal settings for both the *nrg^849^* and the wildtype brains. The intensity of the DenMark signals was measured from a defined region at the level of the suboesophageal ganglion (SOG; area of defined region was kept constant for all samples).

Quantification of Dilp peptide levels in IPCs was done based on fluorescence intensity as described (46).

### Determination of Body Weight

Body weight was determined by weighing individual 3-day-old males. Flies were anaesthetized and collected with chloroform instead of CO_2_ to avoid desiccation. The *nrg^849^* stock was isogenized by outcrossing for three generations to CanS.

### Stress Resistance

For both starvation resistance and oxidative stress resistance, 3-5 day-old adult *nrg^849^* and CanS flies or Dilp2-Gal4/+; UAS-*nrg*RNAi/+ and CyO/+; UAS-*nrg*RNAi/+ sibling controls were used. For the additional starvation resistance experiments, repo-Gal4 was also used, as well as *Sema-1a*RNAi and *PlexA*RNAi. For these two RNAis, a neutral *mCherry* RNAi that was generated in the same vector and inserted into the same genomic site was used as a control. For starvation resistance, flies were kept on 2% agar, and dead flies were scored multiple times per day. For the oxidative stress resistance experiments, flies were kept on 2% agar, 5% sucrose, 20 mM paraquat and scored in bins of 30 minutes using *Drosophila* Activity Monitors (Trikinetics, Waltham, MA, USA). For both stress resistance experiments, flies were housed in an incubator at 25°C on a 12/12-hour on/off light cycle. Resistance to starvation or oxidative stress was defined as the time of death after initiation of food deprivation or paraquat exposure, respectively.

### qRT-PCR Analysis

Sample preparation and qRT-PCR analysis was done as described (46). Briefly, total RNA was extracted from adult heads with TRI reagent (Invitrogen) and reverse transcribed with Transcriptor First Strand Synthesis kit (Roche). qRT-PCR was done using SYBR Green Detection on a StepOnePlus™ Real-Time PCR System. The geometric means of RpS13 and Rp49 were used for normalization. Relative expression levels were calculated with the ΔΔCt method (48). qRT-PCR primer sequences are:

dilp2: Forward GCGAGGAGTATAATCCCGTGAT, Reverse GGATTGAGGGCGTCCAGAT;
dilp3: Forward GCAATGACCAAGAGAACTTTGGA, Reverse GCAGGGAACGGTCTTCGA
dilp5: Forward GAGGCACCTTGGGCCTATTC, Reverse CATGTGGTGAGATTCGGAGCTA
Nrg: Forward ACGATGCACAGGATCAAGGG, Reverse ATCTAAGCCACCGACCTCCT
Sema1a: Forward ACTTTGAAGATATAATCAACGCCCA, Reverse AGCCTGTAAAGAAGCCGACC
PlexA: Forward GGAAAACAAACAGTTTACATGATGG, Reverse TGCGTTGGTGTCATTCTTGC
Dsk: Forward CTGGGCATTAGCCTTCTGCT, Reverse CCTGTAGTCGCCGATCATCC
Rp49: Forward GCCCAAGGGTATCGACAACA, Reverse CTTGCGCTTCTTGGAGGAGA
RpS13: Forward GGGTCTGAAGCCCGACATT, Reverse GGCGACGGCCTTCTTGAT

### Statistical analysis

All statistics were conducted using GraphPad Prism 9.1.1. For assays comparing means, unpaired T-tests for pairwise comparisons were used. Non-parametric results were analyzed using the Mann Whitney U test. For lifespan analysis under oxidative or starvation stress, median lifespan was compared with a log-rank test. For all statistical analyses, we assumed a significance level of 0.05.

## Results

### Neuroglian Is Required for Function and Differentiation of the Insulin-Producing Neurons

**Figure 1:**
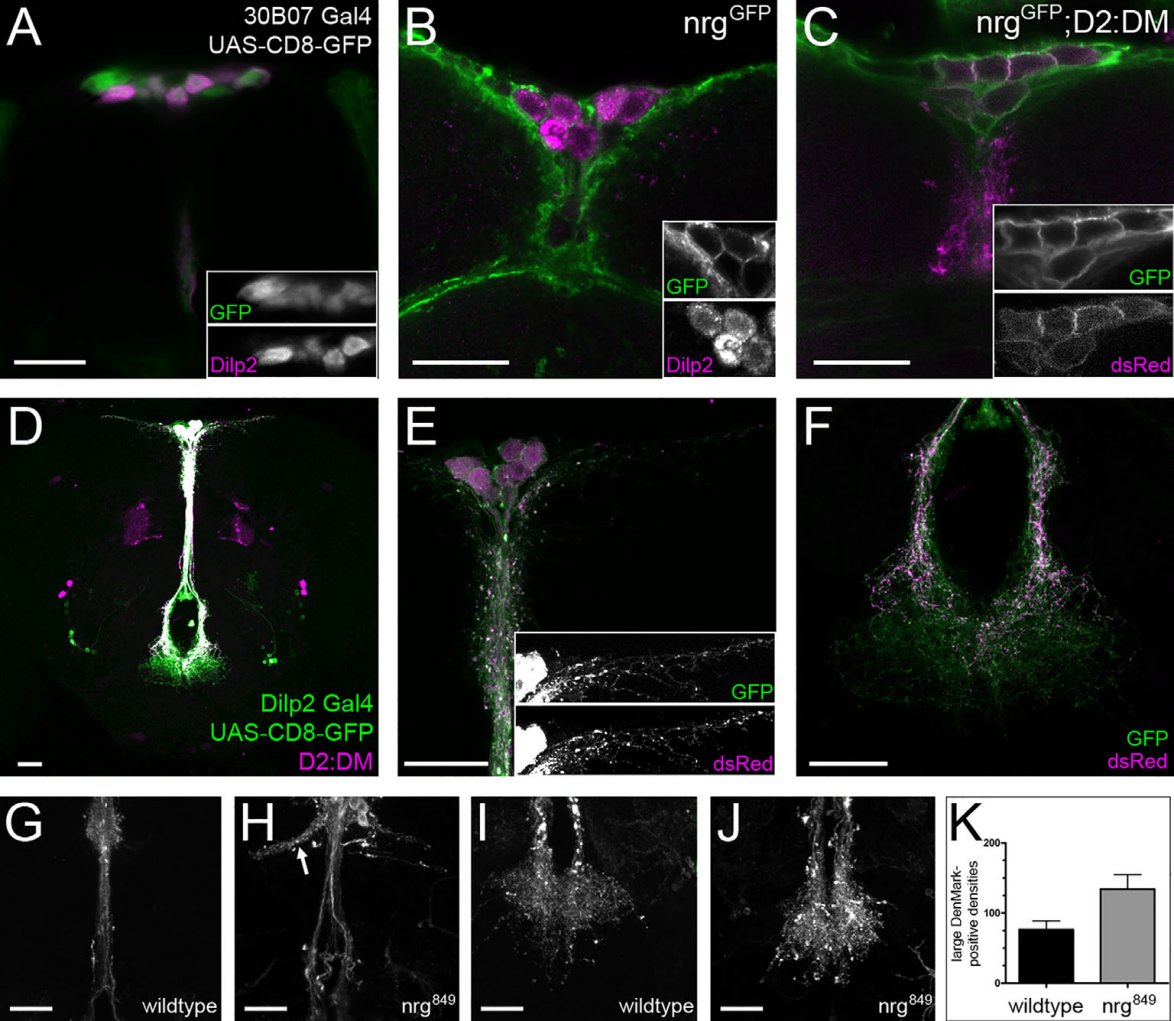
*nrg* is expressed in insulin-producing cells and required for their differentiation. **(A)** The Nrg Gal4 reporter 30B07 is expressed in the IPCs, as shown with double-labeling with anti-GFP (green) and anti-Dilp2 (magenta) **(B, C)** Expression of Nrg (α-GFP, green) can be seen in *nrg^GFP^* brains in the IPCs, labeled with Dilp2 (magenta) as well as with the D2:DM IPC marker (α-dsRed, magenta). **(D–F)** The D2:DM marker (α-dsRed, magenta) is coexpressed in the IPCs with Dilp2 Gal4 > UAS-CD8-GFP (α-GFP, green). **(G, H)** The IPCs of *nrg^849^* mutants display defasciculation and ectopic branching phenotypes (marked by the arrow) as well as **(I-K)** a significant increase in the number of large DenMark-positive densities (unpaired t-test: P=0.027; wildtype, mean = 76.36 ± 12.61, n = 11; *nrg^849^*, mean = 134.1 ± 20.66, n = 11). Adult brains were used in all panels. Scale bars = 20μm.

The insulin-producing cells (IPCs) are a cluster of neurons in the pars intercerebralis of the brain that express Dilps (1, 49, 50). Given that the Nrg enhancer reporters 29H05 and 30B07 drive strong expression in the pars intercerebralis (unpublished observations), we examined whether any of the labelled cells were IPCs. Immunohistochemical analysis revealed that the IPC marker Dilp2 is co-expressed with the 30B07 enhancer reporter (**Figure 1A**). To further validate this finding, we looked for endogenous Nrg expression in the IPCs. Using a GFP-tagged allele of *nrg* (*nrg^GFP^*) (34, 35), we observed clear coexpression of Dilp2 and Neuroglian, confirming that Nrg is expressed in the IPCs (**Figures 1B, C** **)**.

To investigate whether *nrg* is required for normal IPC development, we generated an IPC marker (named D2:DM for Dilp2:DenMark) in which the expression of the somatodendritic marker DenMark is under the direct control of the *dilp2* promoter (1, 40). D2:DM is Gal4-independent, thus enabling us to drive RNAi and rescue constructs in varying cell types without affecting the IPC marker. We observed that the D2:DM marker labels the complete IPC neuron (**Figures 1C–F**) despite DenMark being a selective marker for the somatodendritic compartment of neurons. This indicates that the IPCs dendritic and axonal compartments are not strictly delineated but rather that axoaxonal synapses are formed on the IPCs. We examined the IPCs in *nrg^849^* mutants, a homozygous-viable hypomorph, revealing multiple abnormalities in IPC morphology. One of the strongest phenotypes observed in *nrg* mutants was the strong defasciculation of the neurite projections along the median bundle (**Figures 1G, H** **)**. Additionally, in *nrg* mutants we observe ectopic branches originating from the median bundle, with the ectopic neurites projecting alongside the superior arch commissure (51, 52). These branches form horn-like projections containing a variable number of loosely-associated neurites, which project away from the midline (**Figures 1G, H** **)**. Neurite complexity is also affected in *nrg* mutants, as there are fewer lateral neurites on either side of the soma (**Figure 3B**). In addition, a 75% increase in the number of large DenMark-positive densities within the neurites of the suboesophageal projections is observed (**Figures 1I–K**), suggesting that the distribution of dendritic components and/or synapse formation and remodeling may be altered in *nrg* mutants.

**Figure 2:**
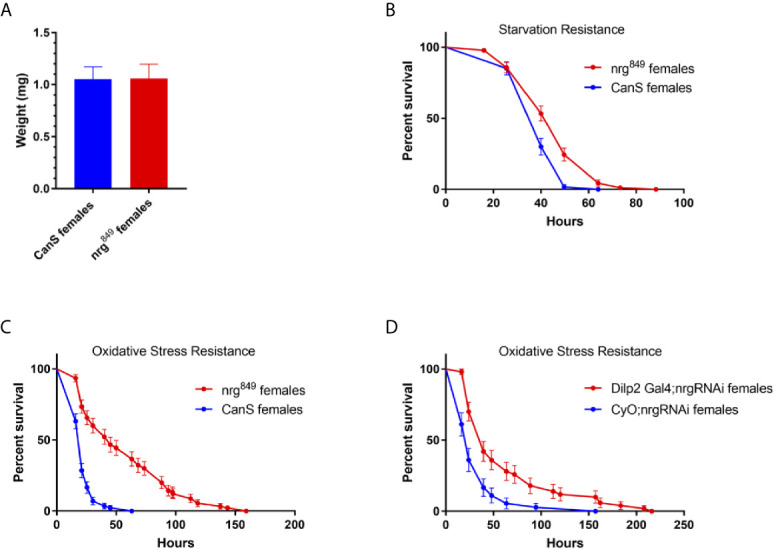
Loss of Nrg results in phenotypes associated with disrupted insulin signaling. **(A)** While there is no difference in *nrg^849^* animal size (P=0.790, Mann Whitney U test), *nrg^849^* mutants are more resistant to **(B)** starvation (P=0.0005, log-rank test; wildtype median survival = 40 hours, *nrg^849^* median survival = 49.75 hours) and **(C)** oxidative stress (P < 0.0001, log-rank test; wildtype median survival = 21 hours, *nrg^849^* median survival = 45 hours). Similarly, IPC-specific knockdown of *nrg* also results in increased resistance to **(D)** oxidative stress (P < 0.0001, log-rank test; wildtype median survival = 24 hours, Dilp2 Gal4:nrg RNAi median survival = 39.5 hours).

No effect on body size of *nrg^849^* mutants was observed (**Figure 2A**), suggesting that the transport and secretion of Dilps, and most prominently Dilp2, to the aorta, where the Dilps are secreted into the circulating hemolymph (1), is not significantly affected. However, we found that *nrg^849^* mutants display other phenotypes associated with insulin signaling in the IPCs, such as enhanced resistance to stress (2, 53). *nrg^849^* mutants show increased resistance to both oxidative stress and starvation (**Figures 2B, C** **)**. Furthermore, these phenotypes persist in flies in which *nrg* is knocked-down specifically in the IPCs (**Figures 2D** and **9A**), consistent with Nrg being required cell-autonomously in the IPCs for normal function.

**Figure 3:**
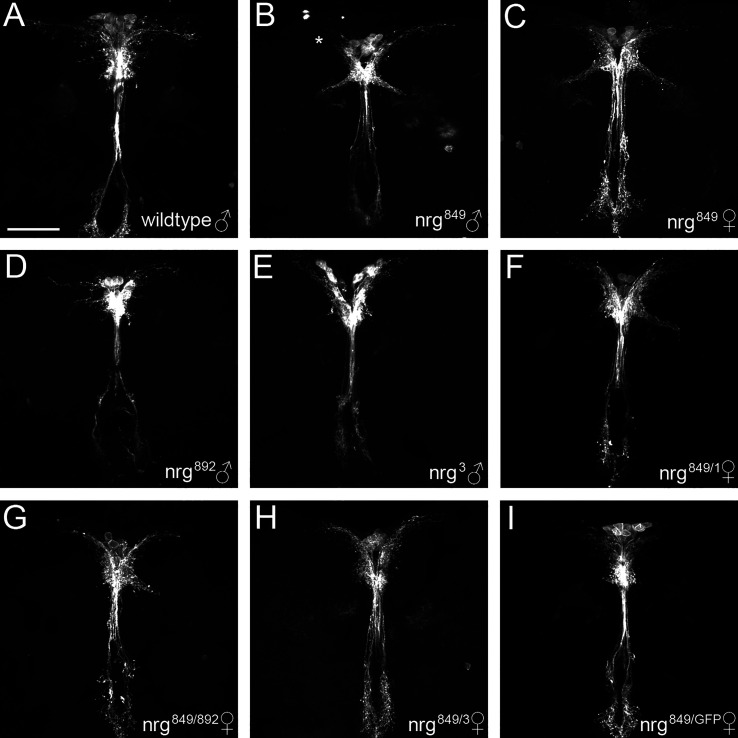
Complementation analysis of *nrg* alleles in IPC neuron development. Compared to **(A)** wildtype adult IPCs, **(B, C)** *nrg^849^* mutants display ectopic branches, median bundle defasciculation, and loss of lateral neurite complexity (asterisk in B). **(D-I)** Similar phenotypes can be seen in *nrg^892^* and *nrg^3^* hemizygous males, and *nrg^849/1^*, *nrg^849/892^*, *nrg^849/3^*, and *nrg^849/GFP^* transheterozygous females. Flies with the temperature-sensitive *nrg^3^* allele were raised at the permissive temperature of 18°C and shifted to the restrictive temperature of 29°C as wandering 3^rd^ instar larvae. IPCs were visualized using Dilp2-GAL4 >GFP-CD8. Scale bar = 50μm.

To investigate whether the phenotypes observed in *nrg^849^* mutants are also seen with other alleles, we analyzed IPC morphology in adults of other allelic combinations of *nrg*, using alleles *nrg^892^*, *nrg^GFP^*, *nrg^3^*, and *nrg^1^. nrg^892^* and *nrg^GFP^* are hypomorphic alleles, *nrg^3^* is a temperature-sensitive allele that behaves as a functional null allele at restrictive temperature, and *nrg^1^* is a homozygous lethal null allele. All allelic combinations examined showed phenotypes similar to what we observed in *nrg^849^*, albeit with variable penetrance (**Figure 3** and **Table 1**). Frequency of ectopic branching was lower in *nrg^892^* and *nrg^3^* hemizygous males (the *nrg* locus is on the X chromosome), as well as in *nrg^849^*/*nrg^3^* females. The observed reduction of lateral neurites was also less penetrant in the three transheterozygous conditions (**Table 1**).

**Table 1:** Complementation analysis of *nrg* alleles.

Genotype	% Ectopic Branches	% Defasciculation	% Sparse Neurites
WT ctrl♂	0 (n=11)	0 (n=11)	0 (n=11)
nrg^849^♂	87 (n=15)	60 (n=15)	73 (n=15)
nrg^849^♀	81 (n=16)	100 (n=15)	56 (n=16)
nrg^892^♂	28 (n=18)	11 (n=18)	72 (n=18)
nrg^3^♂*	23 (n=13)	46 (n=13)	62 (n=13)
nrg^849^/nrg^892^♀	63 (n=8)	57 (n=8)	38 (n=8)
nrg^849^/nrg^3^♀*	38 (n=8)	100 (n=5)	38 (n=8)
nrg^849^/nrg^1^♀	78 (n=18)	89 (n=18)	28 (n=18)
nrg^849^/nrg^GFP^♀	19 (n=16)	19 (n=16)	13 (n=16)

*Flies raised at 18°C and shifted to 29°C as wandering 3^rd^ instar larvae.

**Figure 4:**
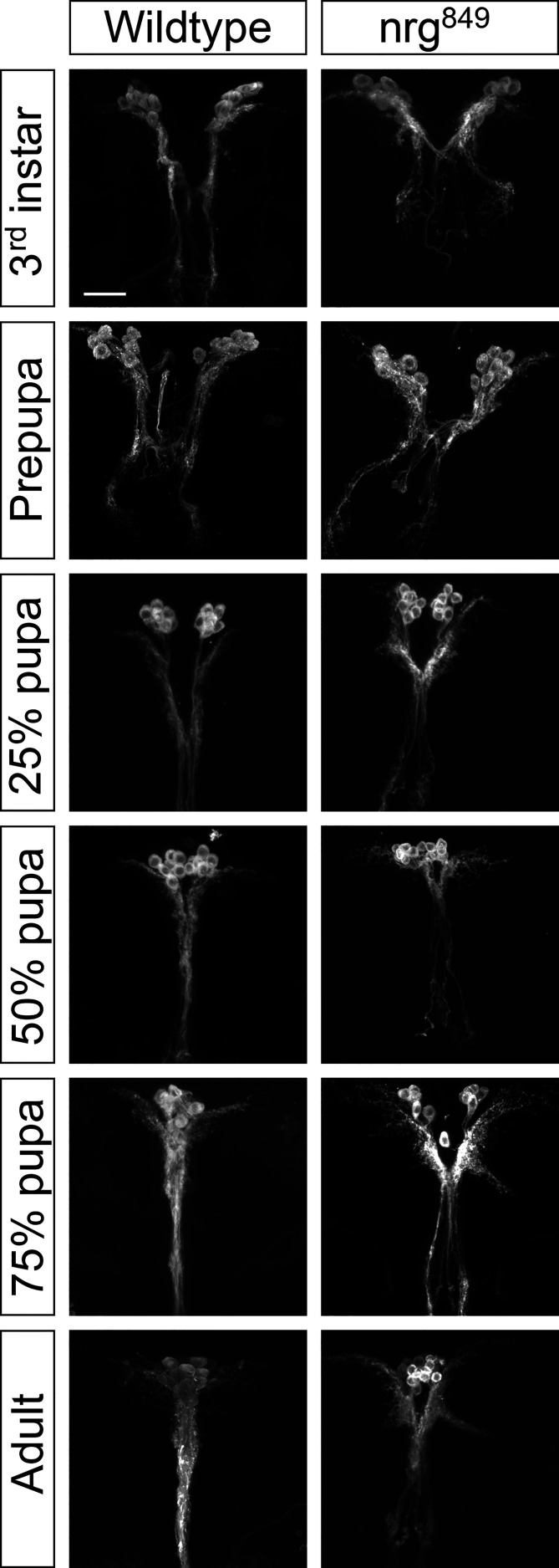
IPC development was studied by imaging the neurons at different stages throughout development (wandering 3^rd^ instar larvae, prepupa, 25% pupa (24 hours after puparium formation (APF)), 50% pupa (48 hours APF), 75% pupa (72 hours APF), and 3-5 day old adults) of flies in which the IPC neurons were labeled *via* UAS-CD8-GFP driven by Dilp2 Gal4. Scale bar = 25μm.

We next examined the development of the IPC neurons throughout late larval and pupal stages to better understand the developmental origin of the *nrg* phenotype. In wildtype larvae, there are two types of prominent IPC neurites: those that project contralaterally and leave the brain to innervate the corpora cardiaca, and those that project ipsilaterally to the subesophageal ganglion (8). While in the larval stage the projections to the corpora cardiaca are outside the brain, these two groups of projections will eventually both project along the median bundle in the adult until they approach the esophageal foramen. In 25% pupae the median bundle has fully extended into the subesophageal ganglion, but the distal neurites of the median bundle projection from each hemisphere are not yet organized into the uniform fan-shape seen in the adult until the end of the pupal stage (see **Figures 1F, I** **)**. In 50% pupae, the cell bodies from the two hemispheres are clustered at the midline and the median bundle fascicles dorsal of the esophagus have completely merged into one median neuropil. Refinement of the median bundle continues into later pupal stages. In *nrg^849^* mutants, a median bundle defasciculation phenotype can be observed from the 25% pupal stage onwards. Elongation of the lateral neurites also begins in early pupae, where proximoventrally of the IPC soma the projections extend laterally from the neuronal fascicle (**Figure 4**, 25% pupa). These neurites also continue to extend and become more elaborate throughout pupal development. Additionally, the clustering of the neurons and the merger of the neurite projections from the two hemispheres is affected in *nrg^849^* animals. Abnormal clustering of cell bodies was seen consistently in adult brains of *nrg^849^* mutant animals (**Figure 4**, Adult). Ectopic neurites are first observed in 75% pupae, and therefore are formed in a later stage of development when all the neurites of a wildtype IPC neuron have already been formed and are in an advanced stage of development (**Figure 4**, 75% pupa). Since no comparable neurite formation is seen during wildtype IPC development, we conclude that this phenotype is the result of ectopic neurite formation, rather than a failure to prune neurites transiently found during development.

### Loss of Glial Neuroglian Leads to Ectopic Branches

**Figure 5:**
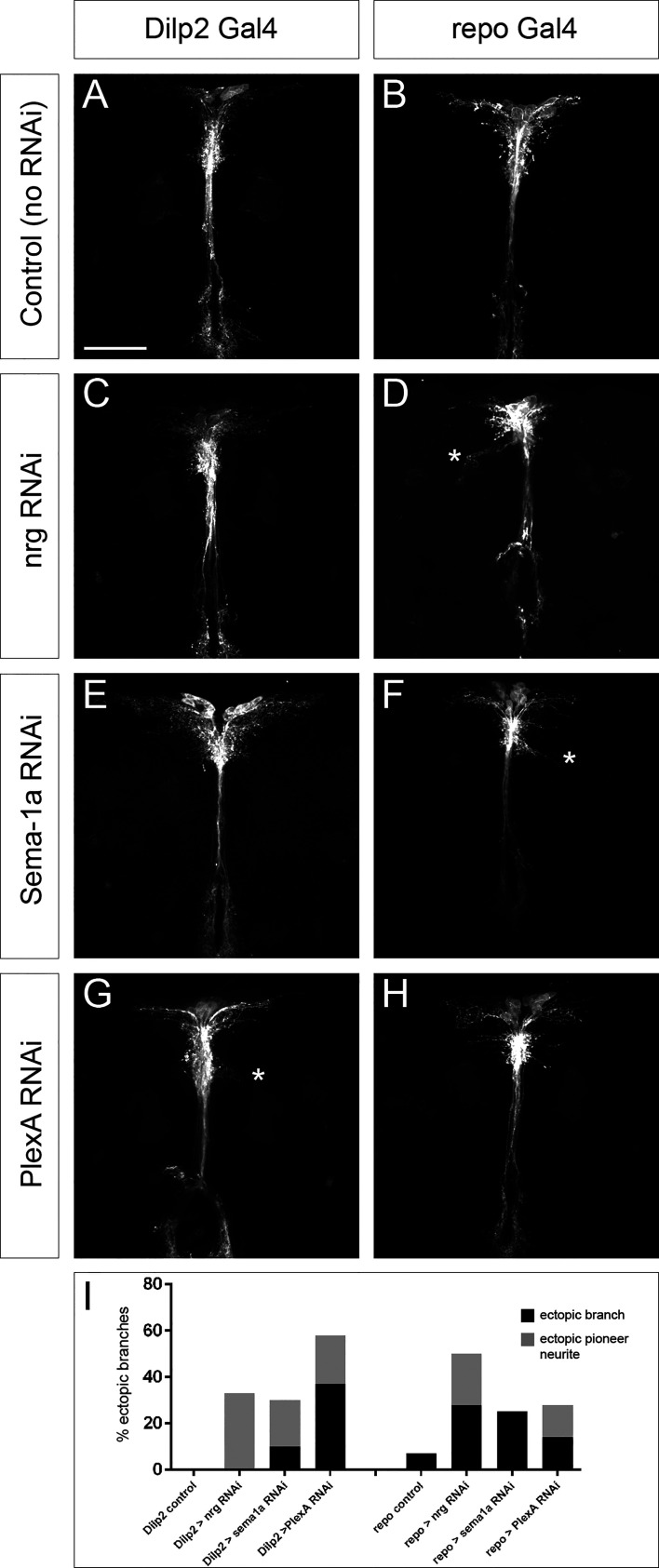
Cell- and non-cell autonomous contributions of *nrg*, *Sema-1a*, and *PlexA* to the IPC neuronal differentiation phenotype. Cell-type specific RNAi knockdowns were performed and compared to **(A, B)** control brains with the Gal4 driver alone. **(C)** Brains in which *nrg* was knocked down in the IPCs do not exhibit any major phenotypes, while **(D)** *nrg* knockdown in glia results in ectopic branching as well as a reduction in lateral neurite complexity. **(E)** IPC knockdown of *Sema-1a* results primarily in an increase in lateral neurite complexity, but also occasionally in mild ectopic branch phenotypes (not shown; see **Table 2**). **(F)** *Sema-1a* knockdown in glial cells results in more robust ectopic branches. **(G)** Knockdown of the Sema-1a receptor *PlexA* in IPC neurons gives rise to ectopic branches with high penetrance, while **(H)** knockdown of *PlexA* in glia results in ectopic branches much less frequently. **(I)** Graph showing the frequency of ectopic branching phenotypes (both ‘mature’ ectopic branches as well as immature single-neurite pioneers) in the different RNAi knockdown conditions. Ectopic branches in **(D, F, G)** are marked with an asterisk. Scale bar = 50μm.

**Figure 6:**
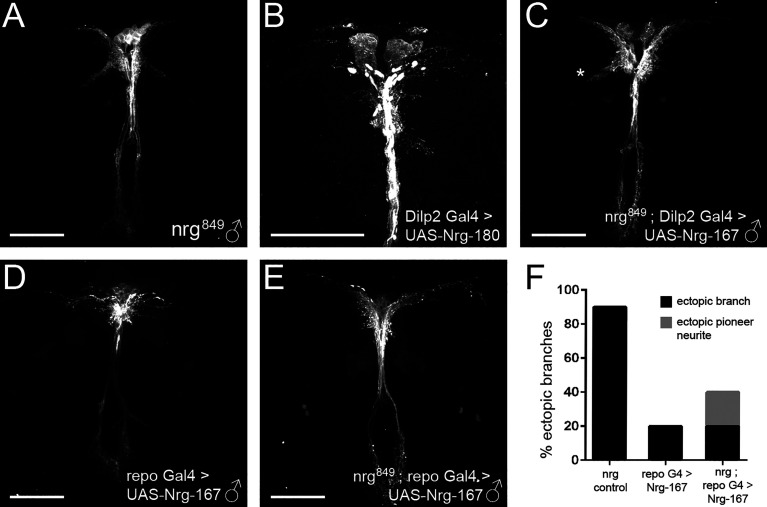
Glial-specific expression of Nrg rescues the ectopic branching phenotype of *nrg^849^* mutants. Attempts to rescue the **(A)** ectopic branch phenotype of *nrg^849^* hemizygous males with **(C)** IPC-specific expression of Nrg failed to rescue the ectopic branching (marked by asterisk). However, **(E, F)** restoration of Nrg-167 expression in glia was sufficient to rescue the ectopic branching phenotype in *nrg^849^* hemizygous males. Overexpression of Nrg in **(B)** IPC neurons or **(D)** glia results in decreases in lateral neurite complexity as well as a redistribution of DenMark. Scale bars = 50μm.

To further investigate the ectopic branching phenotype, we sought to address whether these defects in IPC differentiation were due to a cell-autonomous requirement of Nrg within the IPC neurons themselves. We specifically knocked down *nrg* levels in the IPCs using Dilp2-Gal4 to drive a previously characterized nrg RNAi line that effectively phenocopies loss-of-function mutations (23). Specificity of Dilp2-Gal4 and of efficiency of the Nrg-RNAi was verified using a combination of immunohistochemistry and qRT-PCR (see **Supplemental Figures S1** and **S2**). IPC-specific knockdown of *nrg* did not reproduce any of the ectopic branching phenotypes observed in *nrg^849^* mutants and resulted only in subtle neurite phenotypes (**Figures 5A, C** and **Table 2**). However, in 33% of the brains examined, single ectopic pioneer neurites could be seen (**Figure 5I**). Additionally, neither the IPC-specific expression of Nrg-180 nor Nrg-167 rescued either the ectopic branching or the defasciculation phenotypes in *nrg^849^* mutants (**Figure 6C** and data not shown). Nrg-RNAi mediated knockdown using Dilp2-Gal4 had no impact on IPC cell number (see **Supplemental Figure S3**).

**Table 2:** IPC and glial requirement of Nrg, Sema-1a, and PlexA.

Genotype	% Ectopic Neurites	% Defasciculation	% Sparse Neurites	% Excessive Neurites
Dilp2 Gal4 control	0 (n=13)	0 (n=13)	0 (n=13)	0 (n=13)
Dilp2 Gal4 > nrg RNAi	33 (n=15)	0 (n=15)	13 (n=15)	0 (n=15)
Dilp2 Gal4 > semala RNAi	30 (n=20)	45 (n=20)	5 (n=20)	70 (n=20)
Dilp2 Gal4 > PlexA RNAi	58 (n=19)	42 (n=19)	0 (n=19)	21 (n=19)
repo Gal4 control	7 (n=30)	17 (n=30)	17 (n=30)	0 (n=30)
repo Gal4 > nrg RNAi	50 (n=18)	17 (n=18)	83 (n=18)	0 (n=18)
repo Gal4 > sema1a RNAi	25 (n=12)	8 (n=12)	25 (n=12)	0 (n=12)
repo Gal4 > PlexA RNAi	29 (n=14)	29 (n=14)	7 (n=14)	14 (n=14)

Given our results demonstrating that the observed *nrg^849^* IPC morphological defects are largely non-autonomous and the previously described link between Nrg-mediated neuron-glia interactions and axon sprouting (35, 54), we investigated whether glia-expressed Nrg had any role in IPC differentiation. Using the repo-Gal4 driver to express nrg RNAi, we specifically knocked down *nrg* in glial cells. Specificity of Repo-Gal4 was verified by means of immunohistochemistry (see **Supplemental Figure S1**). Glial loss of *nrg* results in IPCs with ectopic branches, median bundle defasciculation, and aberrant neurite arborization (**Figures 5B, D** and **Table 2**). To further test the requirement of glia-expressed Nrg in IPC ectopic branch formation, we attempted to rescue the ectopic branching phenotype seen in *nrg^849^* hemizygous males by expressing the Nrg-167 isoform with repo-Gal4. Expression of Nrg-167 in glia was sufficient to rescue the ectopic branching phenotype, further demonstrating that Nrg expression in the glial cells surrounding the IPC neurons plays a crucial role in regulating IPC neurite branching (**Figures 6A, E, F** **)**.

In the course of the rescue experiments, we observed that overexpression of the Nrg-180 isoform in wildtype IPCs resulted in a dramatic decrease in lateral neurite branching (**Figure 6B**). Furthermore, there is a clear redistribution of the dendritic DenMark marker in the lateral neurites, where DenMark is predominantly localized proximoventrally of the IPC soma instead of the more diffuse localization observed in wildtype (**Figure 6B**). Similar to the overexpression phenotypes seen in the IPCs, overexpression of Nrg-167 in glia resulted in IPC neurons with reduced lateral neurite complexity as well as aberrant DenMark localization (**Figure 6D**).

### Disruption of Semaphorin-1a/PlexinA Signaling Between IPCs and Glia Results in Ectopic Branches

**Figure 7:**
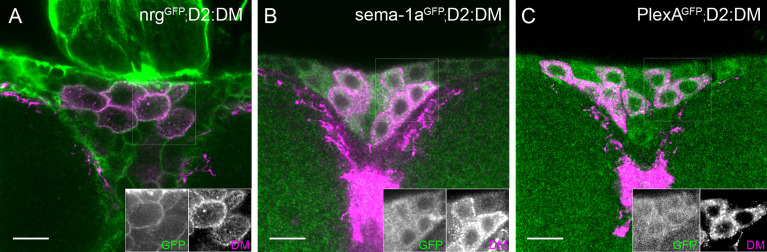
Expression of the signaling molecules Sema-1a and PlexA in the IPCs. Similar to Nrg **(A)**, both Sema-1a **(B)** and PlexA **(C)** are expressed in the IPCs as well as in neighboring cells.

It has been shown that Nrg-mediated axon guidance can be influenced by Sema-1a (23, 54). To determine whether Sema-1a/PlexA signaling might also play a role in IPC neurite pathfinding, we first examined whether they are expressed in the IPCs. Using GFP-tagged alleles, we observed that, like Nrg, both Sema-1a and PlexA are broadly expressed in the pars intercerebralis, with expression in the IPCs as well as in neighboring cells (**Figures 7A–C**). To investigate the role of these molecules in IPC differentiation, we performed targeted knockdown experiments in which an RNAi against either *Sema-1a* or *PlexA* was specifically expressed in the IPC neurons or in glia. Efficiency of RNAi-mediated knockdown was determined using qRT-PCR (see **Supplemental Figure S2**). As with nrg RNAi, all RNAi-Gal4 combinations resulted in IPCs that failed to differentiate properly, suggesting that these three molecules are required in both cell types, consistent with their strong expression in glia and neurons (55). However, IPC knockdown of *PlexA* and glial knockdown of *Sema-1a* resulted in phenotypes much stronger than the other two conditions, indicating that Sema-1a is primarily required in glia, while PlexA is primarily required in the IPC neurons for their proper differentiation (**Figures 5E–I** and **Table 2**). Interestingly, while the ectopic branch phenotype was stronger when *Sema-1a* was knocked down in glial cells, IPC-specific knockdown resulted in an increase of lateral neurite complexity (**Figure 5E** and **Table 2**). Sema1-a and PlexA-RNAi mediated knockdown using Dilp2-Gal4 had no impact on IPC cell number (see **Supplemental Figure S3**).

### Nrg, Sema-1a, and PlexA Influence Stress Resistance

**Figure 8:**
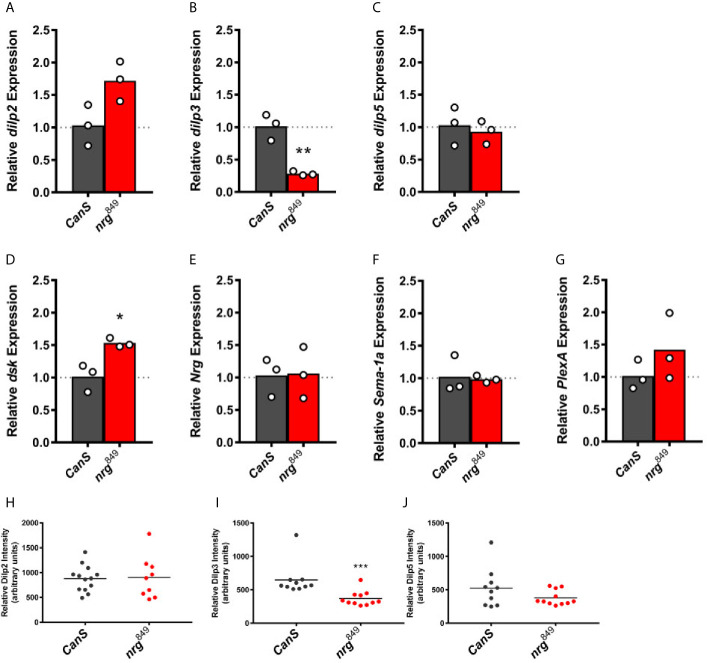
Expression analysis of the IPC-expressed *dilps, dsk, nrg, Sema-1a*, and *PlexA* in *nrg^849^* adult female heads. At the transcript level, expression of *dilp2* **(A)** is elevated 72% (unpaired t-test: P = 0.0530), *dilp3* **(B)** is reduced to 28% of wildtype levels (**P = 0.0034), *dilp5* **(C)** is unchanged, *dsk* **(D)** is elevated 53% (unpaired t-test: *P = 0.0159), and expression of *nrg* **(E)**, *Sema-1a* **(F)**, and *PlexA* **(G)** are unaffected. At the peptide level, revealed by immunohistochemistry, Dilp2 levels **(H)** are unchanged, Dilp3 peptide levels **(I)** are reduced by 43% (unpaired t-test: ***P = 0.0003), and Dilp5 levels **(J)** are unaffected.

In order to better understand how loss of *nrg* in IPCs may affect stress resistance (**Figures 2B–E**), we analyzed how expression of *dilp2*, *dilp3*, and *dilp5*, the three IPC-expressed dilps, *dsk*, which encodes an IPC-expressed peptide involved in feeding behavior (56), as well as *nrg*, *Sema-1a*, and *PlexA* are affected in adult female *nrg^849^* hypomorphs (**Figure 8**). In *nrg* females, *dilp3* expression is significantly reduced to 28% of wildtype levels, and *dsk* expression is increased by 53% (**Figures 8B, D** **)**. We also found *dilp2* expression to be elevated by 72%, which is borderline statistically significant (p=0.053; **Figure 8A**). No significant changes in the expression of *nrg*, *Sema-1a*, or *PlexA* were observed (**Figures 8E–G**). Similarly, Dilp3 peptide levels in the soma of adult female *nrg^849^* mutants are reduced by 43%, while levels of Dilp2 and Dilp5 peptide are not altered (**Figures 8H–J**).

**Figure 9:**
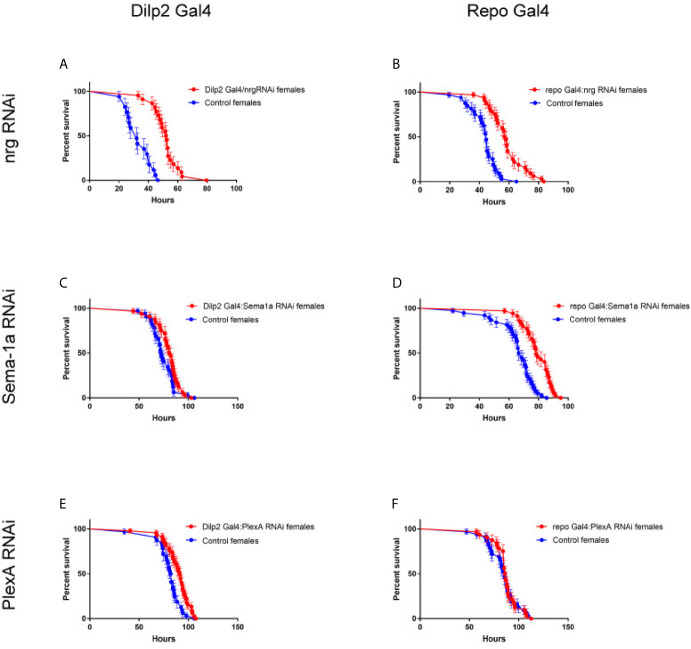
Cell-type specific loss of *nrg, Sema-1a*, and *PlexA* gives rise to starvation stress survival phenotypes. **(A)** Knockdown of *nrg* in IPCs with Dilp2 Gal4 results in increased starvation resistance (IPCs: P<0.0001, log-rank test; control median survival = 32 hours, RNAi median survival = 52.25 hours; **(B)** and in Glia: P<0.0001, log-rank test; control median survival = 44.5 hours, RNAi median survival = 57.5 hours). Knockdown of *Sema-1a* in IPCs **(C)** does not significantly affect survival (P=0.1120, log-rank test; control median survival = 72.25 hours, RNAi median survival = 81.25 hours), while knockdown of *Sema-1a* in glia **(D)** extends survival by 16% (P<0.0001, log-rank test; control median survival = 67.5 hours, RNAi median survival = 78.5 hours). The opposite is seen with *PlexA*, where knockdown of *PlexA* in IPCs **(E)** does extend survival by 11% (P=0.0014, log-rank test; control median survival = 82.25 hours, RNAi median survival = 91.5 hours), while knockdown of *PlexA* in glia **(F)** does not affect survival (P=0.7509, log-rank test; control median survival = 85.5 hours, RNAi median survival = 86.25 hours).

We then expanded the cell-type specific RNAi knockdown experiment to downregulate *nrg*, *Sema-1a*, and *PlexA* in IPCs (Dilp2-Gal4) and glia (repo-Gal4) and investigated the impact on starvation resistance in adult females. Knockdown of *nrg* in either IPCs or glia results in starvation resistance phenotypes similar to what we observed in *nrg^849^* animals, demonstrating that *nrg* is required in IPCs and glia for this phenotype associated with disrupted insulin signaling (**Figures 9A, B** **)**. Knockdown of *Sema-1a* in the IPCs did not have an effect on starvation resistance, while *Sema-1a* knockdown in glial cells did significantly increase starvation resistance (**Figures 9C, D** **)**. Conversely, knockdown of the *PlexA* receptor in the IPCs resulted in an 11% increase in starvation resistance, while knockdown of the receptor in glia had no effect on the phenotype (**Figures 9E, F** **)**.

## Discussion

Here we identify the requirement of three genes, *nrg*, *Sema-1a*, and *PlexA* in the morphological and functional differentiation of IPC neurons, and identify glia as a source of morphoregulators controlling neuritogenesis of the IPCs. When transcript levels of either *nrg* or *Sema-1a* were reduced in glia, ectopic neurite branches formed on the IPC neurons. This phenotype was reproduced when *PlexA*, which encodes the Sema-1a receptor, was knocked down in the IPCs. We also describe a role of Nrg in synaptic organization of the IPCs.

The cell adhesion molecule Neuroglian has been shown to be a key factor in nervous system development, where it is expressed both in neurons and glia. *nrg* is required for IPC development and differentiation, as observed by the severe defasciculation and misbranching phenotypes caused by loss of Nrg function. Furthermore, we show that Nrg is predominantly required in glia for proper IPC neurite formation. When *nrg* was knocked down in glia, we observed the formation of ectopic branches as well as a loss of neurite complexity in the IPC complex, whereas IPC-specific knockdown of *nrg* only produced single pioneer neurites. This suggests that while cell-autonomous Nrg may be necessary for the initial stages of ectopic branch formation, glia-encoded Nrg has a repulsive function in preventing additional neurites from fasciculating onto the pioneer neurites. It is also noteworthy that the developmental stage in which we first observe ectopic branches in *nrg* mutants (75% pupa), corresponds with the CNS-wide assembly of adult neural circuits, a process that is dependent on glia (57). Glia expression of Nrg-167 was able to rescue the ectopic branching phenotype, demonstrating that the glia-encoded Nrg is sufficient to control neurite outgrowth. This is reminiscent of embryonic CNS development, where glia are not absolutely required for pioneer neurite formation, but are necessary for the ensuing fasciculation of follower neurites onto the pioneer (58). Interestingly, ectopic branches have also been observed in the dendritic arborization (da) neurons of *nrg^2^* mutants (35). While expression of the Nrg-167 isoform in da neurons alone failed to rescue the ectopic branching phenotype, expression of Nrg-167 in both da neurons and glia was able to completely rescue the ectopic branching phenotype (35). Although a rescue of the ectopic da branching was not demonstrated by driving Nrg in glia alone, this data supports the model that glia-encoded Nrg acts as a suppressor of neurite sprouting.

In vertebrates, L1-CAM, in a receptor complex with Plexin-A1 and Neuropilin-1, interacts with Semaphorin3A to influence neurite outgrowth and growth cone stability in neuronal cell culture experiments (59–62). In *Drosophila*, Sema-1a has been shown to interact genetically with Nrg (22, 23). In addition to identifying a role for Nrg in IPC differentiation, we demonstrated that Sema-1a and its receptor, PlexA, also are required in IPC differentiation. To our knowledge, this is the first time Sema-1a has been shown to be required in glia to control neurite outgrowth. In vertebrate models, both Sema3A and Sema6D have been shown to be required in glia in the regulation of axonal outgrowth in motor neurons and retinal axons, respectively, presumably *via* PlexA expressed in the developing neuron (63, 64). Interestingly, in the retinal axon model, glial expression of the L1-related Nr-CAM was also required to mediate the effects of Semaphorin (63) Our data suggest that this combination of glia-expressed L1- (or L1-related) CAM and Semaphorin and neuronal PlexA in regulation of neurite outgrowth is evolutionarily conserved.

Despite the major morphological defects observed in the IPCs of *nrg* mutants, at least one essential function of these neurons in insulin signaling appears to be unaffected, as we did not observe any growth defects in *nrg* mutant animals. This observation, as well as the lack of more overt morphological phenotypes upon IPC-specific *nrg* knockdown, could to an extent be due to compensatory effects by other IPC-expressed CAMs, such as Fasciclin 2, which is also expressed in the IPCs (unpublished data and 8) and previously shown to be functionally interchangeable in some axons (65). However, we cannot rule out the possibility that loss of IPC-specific *nrg* results in subtle defects affecting signaling, connectivity, or ultrastructural morphology. Our observations that loss of *nrg* in the mutant as well as in cell-type specific knockdown in both IPCs and glia results in stress resistance phenotypes suggests the existence of one or more of these underlying defects. Loss of *nrg* leads to a strong reduction of Dilp3 (observed at both the mRNA and peptide levels) and an increase in *dsk* expression. While both gene products have clear roles in metabolism, it seems unlikely that they can explain the observed *nrg* phenotypes. Loss of *dilp3* alone has been shown not to have any significant effect on stress resistance, and knockdown of *dsk* has been shown to increase stress resistance (56, 66). It is likely that both gene products, together with Nrg, Sema-1a, PlexA, and other factors all contribute to the phenotype. That loss of *Sema-1a* in glia and *PlexA* in the IPCs also gives rise to altered stress resistance demonstrates that these molecules are also involved in the regulation of IPC function. These phenotypes, taken together with the glia-regulated neurite outgrowth phenotypes, demonstrate a requirement for Nrg in both the IPCs and glia for the proper development and function of these neurons. Furthermore, a comparison of the stress resistance with the neurite outgrowth phenotypes from the cell-type specific RNAi knockdown experiments shows a prominent need for Nrg in both cell types, while the Sema-1a ligand is required in glia, and PlexA, the Sema-1a receptor, in the IPCs. These data suggest that Nrg and Sema-1a in glia work jointly to coordinate IPC neurite outgrowth and function, and this together with IPC-expressed PlexA.

We observed increased numbers of large DenMark-positive densities in the SOG neurites of *nrg^849^* mutants, as well as aberrant localization of DenMark upon overexpression of Nrg-180. Since DenMark is enriched at postsynaptic sites (40), and Nrg has been implicated in synapse organization (22, 54, 67), the observed alterations in DenMark-positive structures suggest that Nrg has an active role in the distribution of synaptic components and/or the establishment or maintenance of synapses in the IPC neurons, similar to what has been shown in other neuronal types (54). The observed redistribution of DenMark in our overexpression experiments (see **Figures 6B, D** **)**, on the one hand, is likely an ankyrin-independent gain-of-function phenotype, similar to what has been observed in wing neurons (37). This hypothesis is supported by the observation that expression of the GPI-anchored isoform of Nrg, which lacks the intracellular ankyrin-binding domain, gives a very similar phenotype in the IPCs (data not shown). On the other hand, the increased number of large DenMark-positive densities in the neurites of *nrg^849^* mutants (see **Figures 1I–K**), is a loss-of-function phenotype and suggests that the organization of the cytoskeleton is altered at these synapses. Aggregated Nrg interacts with Ankyrins, leading to the assembly of the spectrin cytoskeleton (68–70). Intriguingly, when components of the spectrin scaffold are removed postsynaptically, significantly larger active zones are also seen at the larval neuromuscular junction (71). Given the similarity between our observations in the SOG, the data of (71), and the well-established link between Nrg, Ankyrin and the spectrin cytoskeleton, we hypothesize that the increased number of large DenMark-positive densities in the SOG is a result of disrupted spectrin-mediated synapse development. Alternatively, the SOG phenotype may be the result of an Ankyrin-independent mechanism, as was proposed for the observed synaptic defects in the giant fiber of *nrg^849^* mutants (54). Our data with DenMark demonstrate that the localization of synaptic proteins is also likely to be altered in *nrg* mutants.

It has been postulated that the neurosecretory cells in the pars intercerebralis and the ring gland in insects and the endocrine system of vertebrates are derived from a common ancestral system, as they both are derived from anterior neuroectoderm and oral ectoderm and are specified by the same transcription factors (4). Our data builds upon this idea and suggests that L1/Semaphorin/Plexin A-signaling may also have a role in the development as well as the function of vertebrate endocrine systems. In vertebrates, weak expression of L1-CAM is found in adult islets of Langerhans, where the endocrine cells of the pancreas are found, and a single nucleotide polymorphism near the CHL1 locus, another L1 homolog, has been associated with insulin resistance (72, 73). L1-CAM is broadly expressed in neurons of the CNS, and has enriched expression in the hypothalamus, including the supraoptic tract nucleus and in the paraventricular nucleus (PVN), both of which project to the neurohypophysis, and the thalamus, with particularly high expression in the midline thalamic nuclei, which includes the paraventricular thoracic nucleus (tPVN) (74). Semaphorins and Plexins are also found in the hypothalamic-pituitary axis (75). Furthermore, while expression of L1-CAM in the CNS was thought to only be found in neurons, expression in pituicytes, a specialized set of glial cells in the hypophysis, is detected from the moment that the neurohypophysis is innervated (72, 76). These pituicytes interact with the neurosecretory axons of the neurohypophysis and may even play an active role in controlling neurosecretion (77). The PVN has been shown to control the ingestion of carbohydrates, and this role in modulating feeding behavior is mediated by Neuropeptide Y (78, 79). The tPVN also plays a role in feeding behavior, where it can anticipate feeding (80). Interestingly, *dilp* expression is stimulated by sNPF, an ortholog of NPY, in the IPCs, which express the sNPF receptor and seem to contact sNPF-expressing neurons (81, 82). In line with the PVN and tPVN, the IPCs have also been shown to influence feeding behavior (83). These data suggest that L1-type cell adhesion molecules may be required in these processes. In summary, we have shown that Nrg, Sema-1a and PlexA are required in IPCs and glia to control morphological and functional differentiation of IPCs.

## Data Availability Statement

The original contributions presented in the study are included in the article/**Supplementary Material**. Further inquiries can be directed to the corresponding author.

## Author Contributions

JC and PC conceived research project and designed experiments. JC, KB, MW, and VV performed experiments. JC, MW, and PC interpreted data and wrote manuscript. JC, KB, MW, VV and PC revised the manuscript. All authors contributed to the article and approved the submitted version.

## Funding

This work was supported by FWO grants G065408.N10 and G078914N and by KULeuven grant C14/17/099.

## Conflict of Interest

The authors declare that the research was conducted in the absence of any commercial or financial relationships that could be construed as a potential conflict of interest.

## Abbreviations

IPC, insulin-producing cell; Nrg, neuroglian; Sema-1a, semaphorin-1a; PlexA, plexin A; Dilp, *Drosophila* insulin-like peptide.
