# Supplementary material for: Glial and Neuronal Neuroglian, Semaphorin-1a and Plexin A Regulate Morphological and Functional Differentiation of Drosophila Insulin-Producing Cells
Source: Front Endocrinol (Lausanne). 2021 Jul 1;12:600251. doi: 10.3389/fendo.2021.600251 (PMC8281472; doi:10.3389/fendo.2021.600251)
Supplement: Supplementary file 1 [file DataSheet_1.pdf]

Supplementary Materials

Glial and neuronal Neuroglian, Semaphorin-1a and Plexin A regulate morphological and functional differentiation of *Drosophila* insulin-producing cells

Jason Clements, Kurt Buhler, Mattias Winant, Veerle Vulsteke, Patrick Callaerts

Figure S1

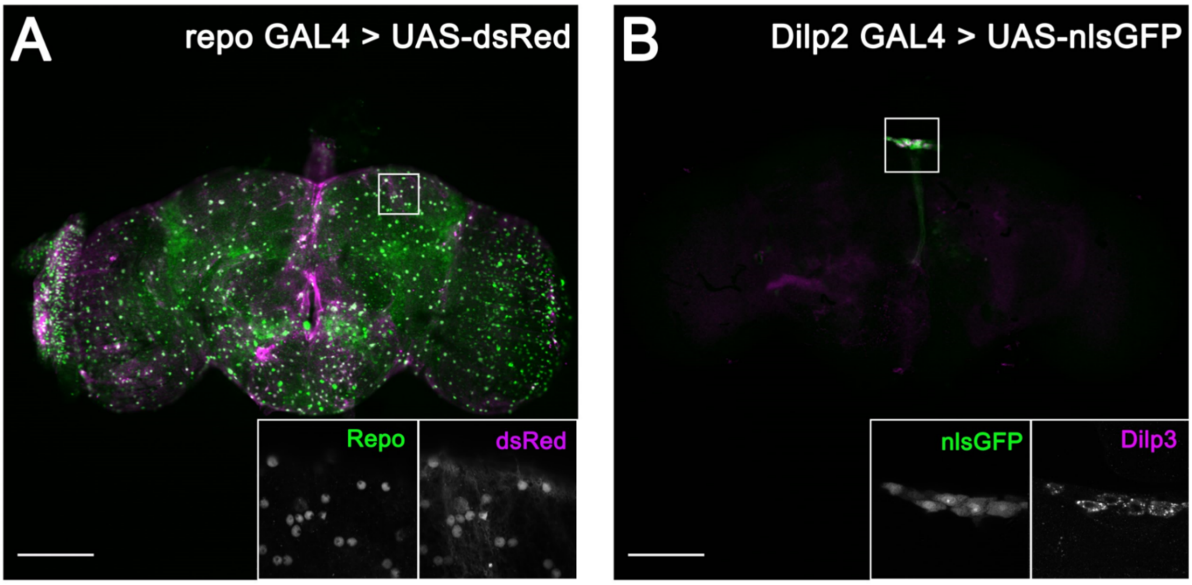

Specificity of GAL4 drivers: (A) Colocalization of Repo protein, a marker protein selectively expressed in glia (green; visualized with primary mouse 8D12 anti-Repo antibody (Developmental Studies Hybridoma Bank, University of Iowa) and FITC-labeled goat anti-mouse antibody, Jackson ImmunoResearch) and dsRed (magenta) expressed under the control of Repo-GAL4. Complete overlap of labeling is observed consistent with Repo-GAL4 being highly selective for glia. (B) Colocalization of Dilp3, a marker for insulin-producing cells (magenta; visualized with primary rabbit anti-Dilp3 antibody and Cy3-labeled donkey anti-rabbit antibody, Jackson ImmunoResearch) with nuclear GFP (nlsGFP) expressed under the control of Dilp2-GAL4. Complete overlap is observed of cells expressing nuclear GFP and the Dilp3-positive large dense core vesicles in the cytoplasm. Scale bar = 50μm.

Figure S2

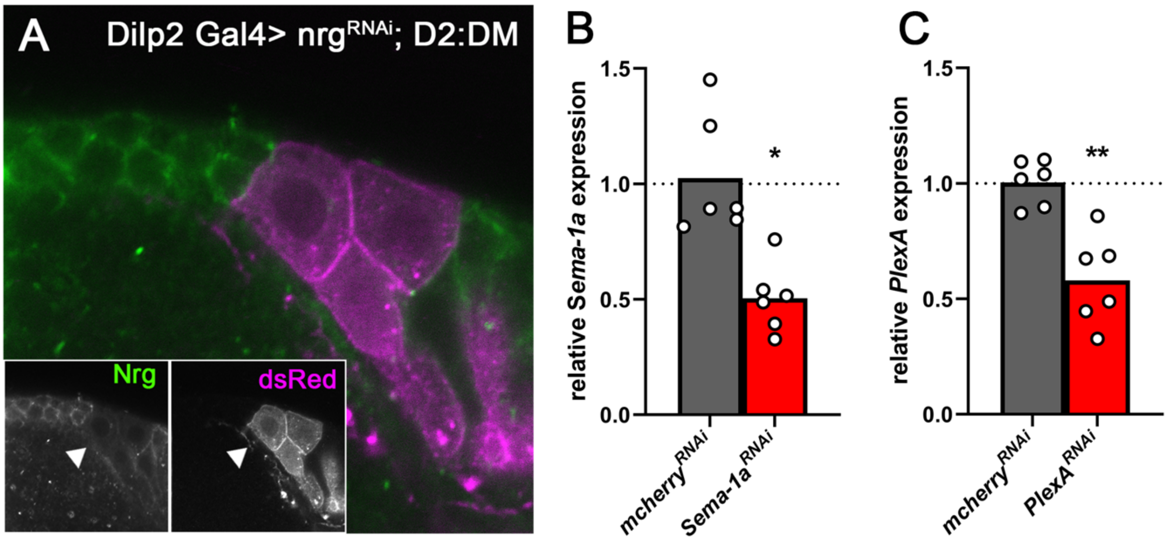

RNAi efficiency: (A) Selective downregulation of Nrg protein (green) using RNAi-mediated knockdown of Nrg in IPCs. D2:DM: Dilp2-enhancer targeted expression of the dsRed-containing somatodendritic marker DenMark in the IPCs. Nrg-RNAi is expressed under the control of Dilp2-GAL4. (B) qRT-PCR to determine Sema-1a RNAi efficiency by comparing expression of Sema-1a relative to the mcherry-RNAi controls. Unpaired T-test, * $p<0.05$. n=6, with each replicate consisting of 3 complete L3 stage larvae expressing Sema-1a RNAi under the control of the ubiquitous Act5C-GAL4 driver. (C) qRT-PCR to determine PlexA RNAi efficiency by comparing expression of PlexA relative to the mcherry-RNAi controls. Unpaired T-test, ** $p<0.01$. n=6, with each replicate consisting of 3 complete L3 stage larvae expressing PlexA RNAi under the control of the ubiquitous Act5C-GAL4 driver.

Figure S3

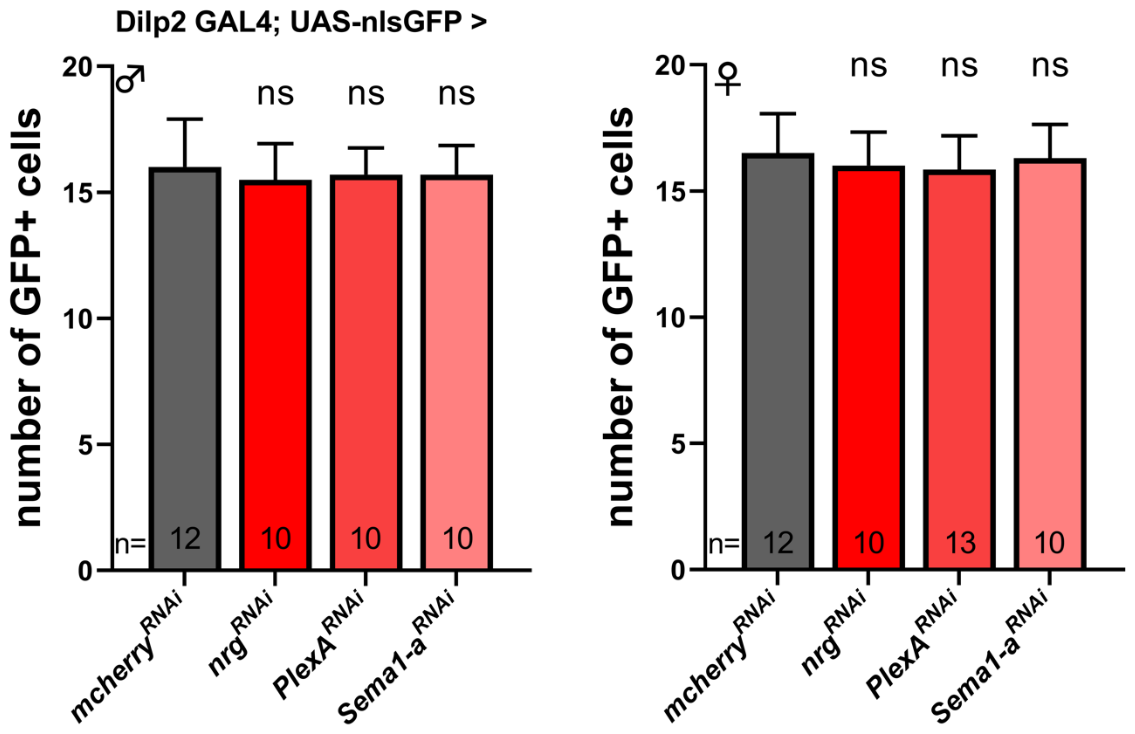

RNAi knockdown has no impact on number of insulin-producing cells: IPCs were visualized using nuclear GFP (nlsGFP) expressed under the control of Dilp2-GAL4. RNAi-mediated knockdown of nrg, PlexA and Sema1-a (also under the control of Dilp2-GAL4) revealed no differences in IPC number compared to mcherry-RNAi controls in males or females. n= number of brains that were analyzed per condition.
